# Associations between Housing Factors and Respiratory Symptoms in Two Saskatchewan First Nations Communities

**DOI:** 10.3390/ijerph18073744

**Published:** 2021-04-03

**Authors:** Naiela Anwar, Shelley Kirychuk, Chandima P. Karunanayake, Vivian Ramsden, Brooke Thompson, Eric Russell, Kathleen McMullin, Donna Rennie, Jeremy Seeseequasis, Mark Fenton, Sylvia Abonyi, Punam Pahwa, James Dosman

**Affiliations:** 1 College of Medicine, University of Saskatchewan, Saskatoon, SK S7N 5E5, Canada; naiela.anwar@usask.ca (N.A.); kathleen.mcmullin@usask.ca (K.M.); 2 Department of Medicine, Canadian Centre for Health and Safety in Agriculture, University of Saskatchewan, Saskatoon, SK S7N 2Z4, Canada; 3 Canadian Centre for Health and Safety in Agriculture, University of Saskatchewan, Saskatoon, SK S7N 2Z4, Canada; cpk646@mail.usask.ca (C.P.K.); brooke.thompson@usask.ca (B.T.); efr443@mail.usask.ca (E.R.); donna.rennie@usask.ca (D.R.); james.dosman@usask.ca (J.D.); 4 Department of Academic Family Medicine, University of Saskatchewan, Saskatoon, SK S7M 3Y5, Canada; viv.ramsden@usask.ca; 5 Community Member, PO Box 96, Duck Lake, SK S0K 1J0, Canada; jccquasis@willowcreehealth.com; 6 Division of Respirology, Critical Care and Sleep Medicine, University of Saskatchewan, Saskatoon, SK S7N 0X8, Canada; mef132@mail.usask.ca; 7 Department of Community Health & Epidemiology, University of Saskatchewan, Saskatoon, SK S7N 2Z4, Canada; sya277@mail.usask.ca (S.A.); pup165@mail.usask.ca (P.P.)

**Keywords:** respiratory health, housing, First Nations, Canada, mold, dampness, smoking, passive smoking, wheeze

## Abstract

Inadequate housing is commonplace in First Nations in Canada, often leading to environmental impacts on housing such as dampness and mold. First Nations communities suffer from a higher prevalence of respiratory-related health conditions than the general Canadian population. There is limited Canadian literature evaluating the relationship between housing factors and the respiratory health of adults within First Nations communities. This study was undertaken with two Saskatchewan First Nations communities. The study population consisted of 293 individuals within 131 households. The individuals completed questionnaires on their general and respiratory health, and one member of each household completed a household questionnaire. The collection of environmental samples from within the house was undertaken. The respiratory outcomes of interest focused on the individuals with ever wheeze, reported by 77.8% of the individuals, and shortness of breath, reported by 52.6% of the individuals. Body mass index, the nontraditional use of tobacco (i.e., current and ex-smoking), the nontraditional use of tobacco in the house (i.e., smoking in the house), dampness in the house in the last 12 months, and always having a smell of mold in the house were significantly associated with respiratory symptoms. The results reveal that respiratory symptom rates were high in the population and housing factors were significantly associated with respiratory symptoms. Addressing and redressing housing inadequacies in First Nations communities are important in preventing additional burdens to health.

## 1. Introduction

First Nations communities suffer from a higher prevalence of respiratory-related health conditions than the general Canadian population [1]. Inadequate housing, including high rates of inadequate housing access, houses in need of major repairs, houses with mold and dampness issues, and overcrowding, is a significant concern in First Nations communities in Canada [2,3,4,5,6,7]. An inadequate infrastructure for housing and maintenance and a lack of affordability for housing further contribute to First Nations’ housing issues [4,5]. These factors are important considerations as contributors to the respiratory health outcomes in First Nations communities.

Respiratory health issues including wheeze [8], bronchitis [9], shortness of breath [10], asthma [11], and chronic obstructive pulmonary disease (COPD) [12] are common in Canadian First Nations populations. Household-level environmental factors including mold [8,10,13,14,15,16], dampness [8,15], indoor environmental tobacco smoke (ETS) [17,18,19,20], overcrowding [2,21], and inadequate ventilation [4,12,19] have been shown to contribute to the respiratory health of general populations. mold. In Canada, having these household environmental factors present is more common in First Nations community houses as compared to general population houses [2,3,4,5,6,7].

There is limited Canadian literature evaluating the relationship between respiratory health and environmental factors present in the house including mold, dampness, crowding, and the nontraditional use of tobacco in the house (smoking). The influence of housing factors on the respiratory health of residents in First Nations communities is important to address and redress. The aim of this study was to evaluate the associations between household environmental factors and respiratory symptoms in adults from two Saskatchewan First Nations communities.

## 2. Materials and Methods

The study focused on data collected from two First Nations communities located in Saskatchewan, Canada. These communities codeveloped and managed a lung health study facilitated by their members. The details of the study approach have been previously published [22]. In brief, the nature and scope of the study evolved through discussions and consultations with the communities. A decision-makers council comprised of elders, band councilors, youth, health directors/health services staff, and housing directors/staff informed the project’s direction, methods, processes, data interpretation, and dissemination. Trained research assistants, who were local First Nations members residing in each community, went door-to-door to explain the project and invite every adult to participate. Data were collected in 2014 as part of Phase 1 of a 5-year longitudinal study focusing on the respiratory health of the study population. The research methods were reviewed and approved by the communities and the University of Saskatchewan Biomedical Research Ethics Board (Bio#12-189).

### 2.1. Survey Questionnaires

The study population consisted of 293 individuals within 131 households. The individuals completed a questionnaire to collect information with validated questions about their general and respiratory health. One member of each household completed the household questionnaires prior to and during the collection of house environmental samples. The questionnaires aimed to provide a better understanding of the respiratory health of the participants and the house environmental factors.

### 2.2. Respiratory Outcomes of Interest: Shortness of Breath and Ever Wheeze

The primary outcomes of interest were self-reported shortness of breath and ever wheeze.

Shortness of breath was defined by an individual’s “Yes/No” response to any one of the questions: “1. Are you troubled by shortness of breath when hurrying on level ground, floors, or walking up a slight hill?”; “2. Do you have to walk slower than people of your age because you are short of breath?”; “3. Do you ever have to stop for breath when walking at your own pace on the level ground or floor?”; “4. Do you ever have to stop for breath after walking about 100 yards/91.4 m (or after a few minutes) on level ground or floors?”; “5. Are you too short of breath to leave the house or breathless when you are dressing or undressing?”.

Ever wheeze was identified by an individual’s “Yes/No” response to any of the questions: “1. Does your chest ever sound wheezy or whistling: 1a. When you have a cold?; 1b. Occasionally apart from colds?; 1c. Most days? 1d. Most nights?; 2. Do you currently have a wheeze?”.

### 2.3. Housing Environmental Factors from the Questionnaire

The housing questionnaire gathered general information including information about the number of occupants residing in the house, the number of rooms in the house, if the nontraditional use of tobacco (i.e., smoking) was permitted in the house, and questions related to dampness, the presence of visible mold, and the presence of a mildew/moldy or musty odor in the house. A community-derived terminology was established for tobacco use. The traditional use of tobacco in the house was tobacco that was utilized in traditional ceremonies. The nontraditional use of tobacco was any tobacco use (smoking) occurring in the house that was not part of the tobacco used in traditional ceremonies.

### 2.4. Settled House-Dust Collection and Beta Glucan Analysis

A Solaris Turbo Plus vacuum cleaner (model: Miele S514, Germany) set to 950 watts was used to obtain dust samples from the main living area of the house. The dust samples were collected according to the International Study of Asthma and Allergies in Childhood (ISSAC) protocol [23]. The samples were collected in Connaught satin collection sample socks that, prior to sample collection, were autoclaved and preweighed on an Ohaus Adventurer top loading scale (AR1530, Sigma Aldrich, St. Louis, MO, USA). For sampling, a labeled sample sock, followed by a crevice collecting tool, were placed over the distal end of the extension tube of the vacuum hose. A clean crevice tool was used for each collection. The carpeted floors had a 2 m^2^ area vacuumed for 4 min, and the smooth floors were vacuumed for 4 min on a 4 m^2^ area. After the sample collection, the sample socks containing the dust samples were placed in labeled bags, stored in a cooler containing ice packs during transportation, and refrigerated until postcollection processing.

After collection, the sample socks were brought to room temperature and weighed in the same room and on the same balance as they were prior to collection. The difference between the pre- and post-weights were calculated to determine the total amount of dust collected. The dust samples were then sieved through a 300 µm mesh sieve (Fisher Scientific, Mississauga, ON, Canada), and the sieved dust was weighed, placed in labeled snap-seal polypropylene containers (VWR International, Mississauga, ON, Canada), and stored at 4 °C until β-glucan analysis. The sieved samples were brought to room temperature and 10 mg were weighed on an MX5 microbalance with a Haug anti-static device (Mettler Toledo, Columbus, OH, USA). The samples were extracted for soluble (1,3)-β-d-glucan using 9 mL of 0.05% Tween 20 (Fisher Scientific, Mississauga, ON, Canada) and 1 mL of 3 M NaOH to give a final concentration of 0.3 M NaOH. The solutions were vortexed and shaken at 325 RPM for 10 min then centrifuged (Sorvall ST 16 R, Thermo Fisher Scientific, Mississauga, ON, Canada) at 1000× *g* for 15 min. Dilutions of the supernatants were made for 1 in 100, 1 in 500, 1 in 1000, and 1 in 2000, and adjusted to the pH range of 6 to 8. The aliquots were stored at −80 °C until the analysis. A 1:2000 dilution was determined as the optimum dilution to run the analysis. The levels of soluble (1,3)-β-d-glucan were measured in duplicate using a Glucatell kit following the Kinetic Onset Time protocol, used according to the manufacturer’s specifications (Associates of Cape Cod Incorporated, Falmouth, MA, USA). Standard curves ranging from 3.125 to 100 pg/mL were prepared and used to determine the concentration of (1,3)-β-d-Glucan in each dust sample. The absorbance was read at 405 nm for a duration of 90 min using the Biotek ELx808 plate reader and Gen5 v 2.06 software (BioTek, Winooski, VT, USA). The soluble β-glucan results were reported in ug/g and ug/m^2^ of dust and were further categorized into low β-glucan (<99 ug/g or <37 ug/m^2^) and high β-glucan (> =99 ug/g or > =37 ug/m^2^), using the 75th percentile of the results.

### 2.5. Statistical Analyses

The data were entered and analyzed using SPSS version 24 (IBM SPSS Statistics for Windows, Armonk, NY, USA). The categorical and continuous data were described as frequency and percent, and means and standard deviations, respectively. Body mass index (BMI) was derived from the participants’ self-reported weight and height data using the standard formula of height (cm) 2/weight (kg). The duration of both the dampness and a moldy smell was further categorized into always-present and less than always-present. A crowding index was calculated by dividing the number of rooms by the number of people in the house and was reported categorically as > 1 or < 1. A univariate analysis was used to test the associations between the risk factors and the presence of shortness of breath and ever wheeze. A multivariable logistic regression modeling technique based on the maximum likelihood was used to test the association between risk factors and the presence of ever wheeze. Univariate associations with *p*-values less than 0.20 were included in the multivariate models. Clustering effects within the households were adjusted using generalized estimating equations. The strength of the association was presented as odds ratio (OR) estimates and 95% confidence intervals (CIs). In all the analyses, a *p*-value less than 0.05 was considered significant. Evidence of collinearity existed between the variables dampness and smell of mold, therefore individual multivariate models were undertaken for each of the variables. A further multivariate analysis was not undertaken for shortness of breath due to the lack of associations with housing factors in the univariate model.

## 3. Results

From the two communities, a total of 293 individuals, 154 (52.6%) females and 139 (47.4%) males, from 131 households, participated in this study. The mean age of the participants was 36.9 (SD 15.1) years. The mean levels of soluble 1,3-β-d-glucan from the dust samples were 38.81 ± 71.72 ug/m^2^ (13.86 ug/m^2^; min 0.12–max 844.30) and 67.13 ± 47.98 ug/g (median 50.96 ug/g; min 6.55–max 238.49).

The respiratory outcomes of interest focused on the individuals with ever wheeze, reported by 77.8% (228/293) of the individuals, and shortness of breath, reported by 52.6% (154/293) of the individuals. The frequency of shortness of breath and ever wheeze with individual and household environmental factors are shown in Table 1 and Table 2, respectively.

In the univariate analysis (Table 1), a shortness of breath was more commonly reported in females than in males (*p* = 0.02), and a significantly greater shortness of breath was reported when individuals lived in houses where the nontraditional use of tobacco occurred in the house (*p* = 0.04).

In the univariate analysis for ever wheeze (Table 2) there were significant differences between body mass index, the nontraditional use of tobacco (current and ex-use), the categorical level of β-glucans, living in houses where the nontraditional use of tobacco occurred in the house, a dampness in the house in the last 12 months, and the frequency of a moldy smell in the house. The categorical β-glucan levels were not a good predictor of effect in this population. This β-glucan effect has been seen with other populations in Saskatchewan and may relate to the types and levels of β-glucan present in the air in the province. Saskatchewan is a relatively dry province with many agricultural activities. As β-glucan is a general measure, it is possible that a large proportion of the β-glucans present in the settled dust samples came from agricultural sources, which may have differential inflammatory effects from non-agriculturally sourced β-glucans.

A multivariate modeling was undertaken for ever wheeze (Table 3). Due to the correlations between the dampness and smell of mold variables (correlation coefficient equal to 0.317), two individual models were fitted: Model I for dampness in the last 12 months and Model II for the frequency of a moldy smell. In both multivariate models, significant risk factors for ever wheeze and always having a moldy smell in the house (Model I), and ever wheeze and signs of dampness in the house in the last 12 months (Model II), included body mass index, the nontraditional use of tobacco (current and ex-smoking), and the housing environmental factor of the in-home nontraditional use of tobacco, while smell of mold and dampness were also significant in their respective models.

## 4. Discussion

This study aimed to determine the influence of housing environmental factors on the respiratory health of two First Nations communities in Saskatchewan, Canada. The results revealed that respiratory symptom rates were high in this population and both individual and housing environmental factors were significantly associated with the respiratory symptoms of shortness of breath and wheeze. Body mass index and the nontraditional use of tobacco (current and ex-smoking) were individual level factors that were significantly associated with ever having wheeze. The nontraditional use of tobacco (i.e., smoking) in the house was associated with both shortness of breath and wheeze. Dampness in the house in the last twelve months and always having a smell of mold in the house were significantly associated with ever having wheeze.

High rates of respiratory symptoms were reported in the study population, with ever wheeze reported by 77.8% (228/293) of the participants and shortness of breath reported by 52.6% (154/293) of participants. These rates were higher than the provincial rates in farming and nonfarming populations (shortness of breath, 29.1% and ever wheeze, 40.6%) [24]. Body mass index (BMI) was a significant risk factor for ever wheeze but not for shortness of breath. The average BMI in the study population was 28.9, which is in the overweight classification. An increased weight has been associated with respiratory problems such as shortness of breath [25] and wheeze [26]. The females in this population had a greater risk for shortness of breath, which is similar to the provincial findings in farm and nonfarm populations [24]. The study population’s nontraditional use of tobacco (i.e., smoking) was 76.1% and is significantly higher than the 2017 provincial smoking average of 17.8% [27]. The nontraditional use of tobacco (i.e., smoking) was a significant individual risk factor for ever wheeze, which is similar to the findings of others [24].

Housing environmental factors including the in-home nontraditional use of tobacco (i.e., smoking in the house), dampness, and always having a smell of mold in the house were important to the respiratory health outcomes of the participants. Smoking in the house has been shown to have a significant influence on air quality in the house [20]. In this population, the nontraditional use of tobacco in the house was a significant risk factor for both shortness of breath and ever wheeze. The rate of nontraditional use of tobacco (i.e., smoking) in the house (45%) is higher than the national average of 3.9% [28]. Cigarette smoke exposure, whether passive or active, has been associated with a higher risk of wheeze [18,29] and shortness of breath [17,29,30,31], which is consistent with our results.

These findings revealed that 59% of houses had signs of water or dampness and dampness in the last 12 months, and this dampness was associated with twice the risk of ever wheeze. Previous studies have shown an increased risk for wheeze associated with homes with water damage [8,32] and any dampness [32]. Dampness has been shown to be a risk factor for respiratory conditions. That almost 60% of houses had some form of dampness in the last 12 months strengthens the need to address and redress the houses in need of repair within these communities.

These findings reveal that 48% of the houses reported a moldy smell and 34.5% of the houses always had a moldy smell. Always having a moldy smell in the house was associated with twice the risk of ever wheeze. Multiple studies have found associations between mold and respiratory symptoms [14,32,33,34,35,36,37,38]. A study carried out in one First Nations community in British Columbia, Canada found associations between individuals seeking respiratory treatment and their household mold concentration levels, and strong associations between household levels of dampness and the presence of wheeze and cough in individuals [38]. A meta-analysis found the presence of mold to be associated with wheeze, cough, and asthma [8]. In this study, visible mold was not shown to be a significant risk factor for respiratory outcomes. However, always having a smell of mold in the house was a risk factor for ever wheeze. This relationship between mold odor and wheeze is consistent with the findings of others [8]. The odor produced by microbial volatile organic compounds (MVOCs) from fungi and bacteria [39,40] have failed to show consistent associations with visible mold [41,42,43,44,45,46,47]. However, the odor from MVOCs has been shown to be influenced by cigarette smoke [48,49]. The in-home nontraditional use of tobacco in these homes was high and could be a potential contributing influence to MVOCs and the smell of mold. Further research is required to elucidate the potential associations between the smell of mold, the nontraditional use of tobacco in the house, and respiratory conditions.

The many strengths of this study included the research process that was co-created and co-implemented by academic and community researchers. The results contributed to the limited community- and population-level data available in Canada. The study included both self-reported and objective measures of home environmental factors. There was strong community participation. The tools and applications in this research are transferrable. Several limitations also exist in the study. These were cross-sectional observations and causations cannot be determined. The respiratory symptoms and housing factors were self-reported, and a recall bias may exist.

## 5. Conclusions

These results reveal an increased burden on the respiratory health of this population in which the house was an important contributor. Dampness in the house and always having a moldy smell in the house were significant contributors to the risk of wheeze. Living in houses where the nontraditional use of tobacco (i.e., smoking) occurred in the house, enhanced the risk of shortness of breath and wheeze in participants. Individual factors, including the nontraditional use of tobacco (i.e., smoking), were important contributors to shortness of breath and wheeze. The World Health Organization has stated that addressing housing inadequacy is important for the prevention of inequitable burdens of disease [50]. Addressing housing inadequacies in First Nations communities is important in advancing health equity.

## Figures and Tables

**Table 1:** The individual and housing factors stratified according to the presence/absence of shortness of breath and the unadjusted odds ratio (OR) estimates with 95% confidence intervals (CI).

Variables	Total	Shortness of Breath		Unadjusted OR (95% CI)	*p*-Value
	*n* (%) Mean ± SD	Yes *n* (%)	No *n* (%)		
Sex					
Male	139 (47.4)	63 (40.9)	76 (54.7)	0.57 (0.35, 0.92)	0.02
Female	154 (52.6)	91 (59.1)	63 (45.3)	1.00	
**Age (years)**	36.9 ± 15.1	38.4 ± 16.2	35.4 ± 13.6	1.01 (0.99, 1.03)	0.06
**Body mass index (BMI)**	28.9 ± 15.1	29.2 ± 7.5	28.6 ± 6.4	1.02 (0.99, 1.05)	0.29
**Smoking status**					
Current smoker	223 (76.1)	125 (81.2)	98 (70.5)	1.46 (0.65, 3.28)	0.36
Ex-smoker	38 (13.0)	15 (9.7)	23 (16.5)	0.81 (0.28, 2.28)	0.69
Nonsmoker	32 (10.9)	14 (9.1)	18 (12.9)	1.00	
**In-home nontraditional use of tobacco**					
Yes	132 (45.1)	79 (51.3)	53 (38.1)	1.73 (1.03, 2.88)	0.04
No	161 (54.9)	75 (48.7)	86 (61.9)	1.00	
**Crowding**					
People/room > 1	100 (34.1)	52 (33.8)	48 (34.5)	1.05 (0.59, 1.87)	0.86
People/room < = 1	193 (65.9)	102 (66.2)	91 (65.5)	1.00	
**β-glucan (ug/g)**					
Low (<99)	219 (74.7)	112 (72.7)	107 (77.0)	1.00	
High (≥99)	74 (25.3)	42 (27.3)	32 (23.0)	1.35 (0.74, 2.48)	0.33
**β-glucan (ug/m^2^)**					
Low (<37)	217 (74.1)	109 (70.8)	108 (77.7)	1.00	
High (≥37)	76 (25.9)	45 (29.2)	31 (22.3)	1.34 (0.74, 2.43)	0.33
**Temperature (°C)**	22.8 ± 2.0	22.8 ± 1.9	22.9 ± 2.1	0.99 (0.89, 1.12)	0.98
**Relative humidity (%)**	33.5 ± 9.0	34.1 ± 9.2	32.8 ± 8.8	1.01 (0.99, 1.04)	0.22
**Dampness in last 12 months**					
Yes	172 (58.7)	90 (58.4)	82 (59.0)	0.93 (0.55, 1.56)	0.78
No	121 (41.3)	64 (41.6)	57 (41.0)	1.00	
**Frequency of dampness**					
Always	52 (17.1)	26 (16.9)	26 (18.7)	0.88 (0.51, 1.54)	0.66
Frequently and less	241 (82.3)	128 (83.1)	113 (81.3)	1.00	
**Smell of mold**					
Yes	140 (47.8)	72 (46.8)	68 (48.9)	0.97 (0.59, 1.59)	0.90
No	153 (52.2)	82 (53.2)	71 (51.1)	1.00	
**Frequency of moldy smell**					
Always	101 (34.5)	98 (63.6)	94 (67.6)	1.21 (0.72, 2.01)	0.47
Less than 6 months/year	192 (65.5)	56 (36.4)	45 (32.4)	1.00	
**Visible mold**					
Yes	156 (53.2)	123 (53.9)	33 (50.8)	1.03 (0.62, 1.71)	0.91
No	137 (46.8)	105 (46.1)	32 (49.2)	1.00	

**Table 2:** The individual and housing factors stratified according to the presence/absence of ever wheeze and the unadjusted odds ratio estimates with 95% confidence intervals.

Variables	Total	Ever Wheeze		Unadjusted OR (95% CI)	*p*-Value
	*n* (%) Mean ± SD	Yes *n* (%)	No *n* (%)		
**Sex**					
Male	139 (47.4)	109 (47.8)	30 (46.2)	1.11 (0.65, 1.91)	0.69
Female	154 (52.6)	119 (52.2)	35 (53.8)	1.00	
**Age (years)**	36.9 ± 15.1	36.6 ± 14.2	38.2 ± 17.9	0.99 (0.97, 1.01)	0.41
**Body mass index (BMI)**	28.9 ± 15.1	29.3 ± 7.2	27.6 ± 6.0	1.04 (1.00, 1.08)	0.05
**Smoking status**					
Current smoker	223 (76.1)	184 (80.7)	39 (60.0)	4.80 (2.32, 9.94)	0.001
Ex-smoker	38 (13.0)	29 (12.7)	9 (13.8)	3.57 (1.34, 9.46)	0.011
Nonsmoker	32 (10.9)	15 (6.6)	17 (26.2)	1.00	
**In-home nontraditional use of tobacco**					
Yes	132 (45.1)	117 (51.3)	15 (23.1)	3.46 (1.80, 6.65)	0.001
No	161 (54.9)	111 (48.7)	50 (76.9)		
**Crowding**					
People/room > 1	100 (34.1)	75 (32.9)	25 (38.5)	0.83 (0.43, 1.59)	0.58
People/room < = 1	193 (65.9)	153 (67.1)	40 (61.5)	1.00	
**β-glucan (ug/g)**					
Low (<99)	219 (74.7)	178 (78.1)	41 (63.1)	1.00	
High (≥99)	74 (25.3)	50 (21.9)	24 (36.9)	0.49 (0.26, 0.93)	0.03
**β-glucan (ug/m^2^)**					
Low (<37)	217 (74.1)	165 (72.4)	52 (80.0)	1.00	
High (≥37)	76 (25.9)	63 (27.6)	13 (20.0)	1.47 (0.67, 3.23)	0.33
Temperature (°C)	22.8 ± 2.0	22.9 ± 1.8	22.8 ± 2.3	1.02 (0.87, 1.19)	0.80
Relative humidity (%)	33.5 ± 9.0	33.2 ± 7.7	34.4 ± 12.5	0.98 (0.96, 1.01)	0.23
**Dampness in last 12 months**					
Yes	172 (58.7)	140 (61.5)	32 (49.2)	1.67 (0.89, 3.12)	0.11
No	121 (41.3)	88 (38.6)	33 (50.8)		
**Frequency of dampness**					
Always	52 (17.1)	41 (18.0)	11 (16.9)	1.01 (0.41, 2.48)	0.99
Frequently and less	241 (82.3)	187 (82.0)	54 (83.1)		
**Smell of mold**					
Yes	140 (47.8)	112 (49.1)	28 (43.1)	1.23 (0.66, 2.32)	0.51
No	153 (52.2)	116 (50.9)	37 (56.9)		
**Frequency of moldy smell**					
Always	101 (34.5)	88 (38.6)	13 (20.0)	2.34 (1.11, 4.93)	0.03
Less than 6 months/year	192 (65.5)	140 (61.4)	52 (80.0)		
**Visible mold**					
Yes	156 (53.2)	123 (53.9)	33 (50.8)	1.23 (0.65, 2.31)	0.51
No	137 (46.8)	105 (46.1)	32 (49.2)		

**Table 3:** The adjusted odds ratios (OR_adj_) and their 95% confidence interval (95% CI) based on the multivariate analysis of ever wheeze.

	Model I Smell of Mold OR_adj_ (95% CI)	Model II Dampness OR_adj_ (95% CI)
Age of individual	0.99 (0.96, 1.01)	0.98 (0.96, 1.01)
Sex (reference category, female)	1.18 (0.60, 2.32)	1.16 (0.59, 2.28)
BMI	1.08 (1.02, 1.14) *	1.09 (1.03, 1.15) *
Smoking status		
Current	7.09 (3.16, 15.92) **	7.10 (3.19, 15.77) **
Ex-smoker	5.03 (1.62, 15.59) **	4.69 (1.68, 13.11) *
Nonsmoker (reference category)	1.00	
In-home nontraditional use of tobacco (reference category, no)	3.05 (1.50, 6.21) **	3.57 (1.83, 6.96) **
Dampness in the last 12 months (reference category, no)	-	2.00 (1.09, 3.68) *
Frequency of moldy smell		
Always	2.20 (1.03, 4.69) *	-
Less than 6 months/year (reference category)	1.00	

** *p* < 0.001; * *p* < 0.05.

## Data Availability

Data available upon request. The data presented in this study are available on request from the corresponding author.
